# A synthesis of bacterial and archaeal phenotypic trait data

**DOI:** 10.1038/s41597-020-0497-4

**Published:** 2020-06-05

**Authors:** Joshua S. Madin, Daniel A. Nielsen, Maria Brbic, Ross Corkrey, David Danko, Kyle Edwards, Martin K. M. Engqvist, Noah Fierer, Jemma L. Geoghegan, Michael Gillings, Nikos C. Kyrpides, Elena Litchman, Christopher E. Mason, Lisa Moore, Søren L. Nielsen, Ian T. Paulsen, Nathan D. Price, T. B. K. Reddy, Matthew A. Richards, Eduardo P. C. Rocha, Thomas M. Schmidt, Heba Shaaban, Maulik Shukla, Fran Supek, Sasha G. Tetu, Sara Vieira-Silva, Alice R. Wattam, David A. Westfall, Mark Westoby

**Affiliations:** 1 Hawai’i Institute of Marine Biology, University of Hawai’i at Mānoa, Kāne’ohe, HI 96744 USA; 2 Department of Biological Sciences, Macquarie University, Sydney, NSW 2109 Australia; 3 Division of Electronics, Rudjer Boskovic Institute, Zagreb, Croatia; 4 Department of Computer Science, Stanford University, Stanford, CA USA; 5 Tasmanian Institute of Agriculture, University of Tasmania, Hobart, TAS 7005 Australia; 6 Department of Physiology and Biophysics, Weill Cornell Medicine, New York, NY 10065 USA; 7 Department of Oceanography, University of Hawai’i at Mānoa, Honolulu, HI 96822 USA; 8 Department of Biology and Biological Engineering, Chalmers University of Technology, Gothenburg, SE-412 96 Sweden; 9 Department of Ecology and Evolutionary Biology, University of Colorado, Boulder, CO 80309 USA; 10 Environmental Genomics and Systems Biology Division, Lawrence Berkeley National Laboratory, Berkeley, CA 94720 USA; 11 Department of Energy, Joint Genome Institute, Berkeley, CA 94720 USA; 12 Kellogg Biological Station and Department of Integrative Biology, Michigan State University, East Lansing, MI 48824 USA; 13 Department of Molecular Sciences, Macquarie University, Sydney, NSW 2109 Australia; 14 Department of Science and Environment, Roskilde University, Roskilde, Denmark; 15 Institute for Systems Biology, 401 Terry Ave N, Seattle, WA 98109 USA; 16 Microbial Evolutionary Genomics, Institut Pasteur, CNRS UMR3525, 28 rue Dr. Roux, 75015 Paris, France; 17 Department of Ecology & Evolutionary Biology, University of Michigan, Ann Arbor, MI 48109 USA; 18 Computing, Environment and Life Sciences, Argonne National Laboratory, Argonne, Illinois USA; 19 Institute for Research in Biomedicine (IRB Barcelona), The Barcelona Institute of Science and Technology, Barcelona, 08028 Spain; 20 Catalan Institution for Research and Advanced Studies (ICREA), Barcelona, 08010 Spain; 21 Laboratory of Molecular Bacteriology, Department of Microbiology and Immunology, Rega Institute, KU Leuven Leuven, Belgium; 22 Biocomplexity Institute and Initiative, University of Virginia, Charlottesville, VA 22904 USA

**Keywords:** Microbial ecology, Archaea, Bacteria

## Abstract

A synthesis of phenotypic and quantitative genomic traits is provided for bacteria and archaea, in the form of a scripted, reproducible workflow that standardizes and merges 26 sources. The resulting unified dataset covers 14 phenotypic traits, 5 quantitative genomic traits, and 4 environmental characteristics for approximately 170,000 strain-level and 15,000 species-aggregated records. It spans all habitats including soils, marine and fresh waters and sediments, host-associated and thermal. Trait data can find use in clarifying major dimensions of ecological strategy variation across species. They can also be used in conjunction with species and abundance sampling to characterize trait mixtures in communities and responses of traits along environmental gradients.

## Abstract (summary)

**Table:** 

Measurement(s)	Trait • phenotypic trait • quantitative genomic trait
Technology Type(s)	digital curation
Factor Type(s)	habitat • species
Sample Characteristic - Organism	Archaea • Bacteria
Sample Characteristic - Location	global

Machine-accessible metadata file describing the reported data: 10.6084/m9.figshare.12221732

## Background & Summary

Several research groups have advocated for a trait-based approach to ecology of bacteria and archaea^1–9^, but so far this has remained at the level of conceptual discussion or interpretation of particular study systems. Here we describe a scripted workflow that generates a unified microbial trait dataset suitable for investigating which traits are correlated across species versus which vary independently. The dataset spans the full range of bacterial and archaeal habitats, including fresh and marine waters, soils and sediments, animal and plant hosts, and thermal environments. Data sources include well-established repositories, such as GenBank^10^, Bergey’s Manual of Systematics of Archaea and Bacteria^11^, and a number of compilations published in the literature (Online-only Table 1).

We believe this data product will prove useful to other research groups in several ways. Some may use the current version of the dataset for their own data analyses. They may adjust the scripted workflow to adopt different merger rules; for example, about how data sources are aggregated or prioritized when multiple records are available. Some may choose to update the dataset, since among the contributing data sources several are continuing to receive new data. Some may choose to add further data sources or merge their own data sources, which should be made easier by the scripted structure we provide. Once scripted into the workflow, new or updated data sources can be merged with the current data product in GitHub resulting in a new version of the data product.

Trait data can have a variety of research purposes. Correlations among traits can be investigated to elucidate the main dimensions of variation across species^12^. Species lists and their abundances in communities can be interpreted, for example whether communities have similar trait mixtures despite different taxonomy. Responses of traits along environmental or geographical gradients can be described^13^. If relevant traits are available to combine with species identifications and abundances, aspects of ecosystem function can be inferred.

Synthesizing trait data is a continuing process rather than a finite project. During the time taken to add any particular data source to the merger, new data sources continue to appear. The data merger in its current form and as reported here emphasizes quantitative genomic traits (such as genome size and number of rRNA gene copies) and phenotypic traits (such as potential rate of increase, cell radial diameter and growth temperature).

We have included information from culture on metabolic pathways and carbon substrates. However, we have not yet included metabolic pathways inferred from genomes, and consequently the question of reconciling genome-inferred pathways with culture-observed pathways does not arise. Also we have not yet included presence or absence of specific genes as qualitative traits, for a combination of reasons. First, there are potentially a very large number of such traits. Second, the number of complete genomes available continues to increase rapidly, and so such data will be out of date quickly. Third, there exist a number of databases (MIST^14^, MACADAM^15^, ANNOTREE^16^ for example, and more emerging all the time) that specialize in annotations from genomes. When users wish to ask questions involving these genome-derived traits it will be better for them to link those databases to ours, which can be done using NCBI Taxon IDs.

## Methods

**Fig. 1:**
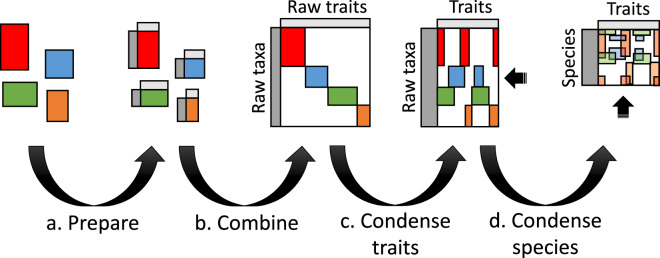
A visual representation of the microbe trait data integration workflow for four hypothetical datasets (red, blue, green and orange). Grey bands represent consistent taxonomy and trait detail that applies across the datasets. Each of the four steps—(**a**) prepare, (**b**) combine, (**c**) condense traits and (**d**) condense to NCBI species—are summarised in the Methods and explained in detail along with scripted steps in R at the GitHub repository.

**Fig. 2:**
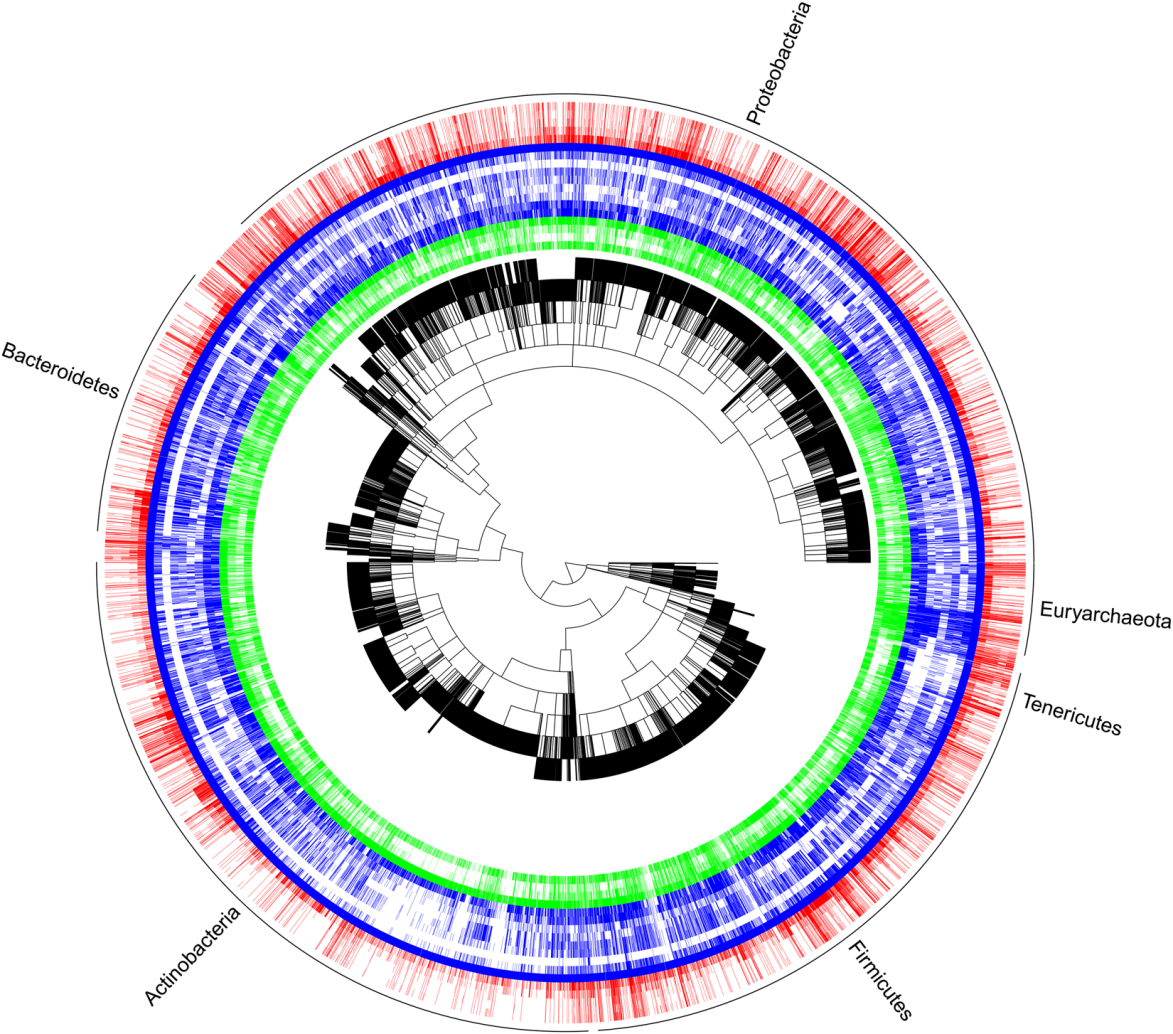
A graphical representation of data coverage and gaps for the 21 core traits mapped onto a phylogeny (black tree). The phylogeny was created by grafting star phylogenies (NCBI species to phylum) onto a recent molecular phylogeny^20^ (phylum and above) and was created here purely for illustrative purposes. To avoid clutter, only the six most speciose phyla are delineated at the outer rim (>100 species). Coloured bands represent the presence of traits in the dataset for 14,884 species. In order for the centre outwards, green are habitat traits (isolation source, optimum pH, optimum temperature, growth temperature), blue are organism trait (gram stain, metabolism, metabolic pathways, carbon substrate, sporulation, motility, doubling time, cell shape, any cell diameter), and red are genomic traits (genome size, GC content, coding genes, rRNA16S genes, tRNA genes).

**Fig. 3:**
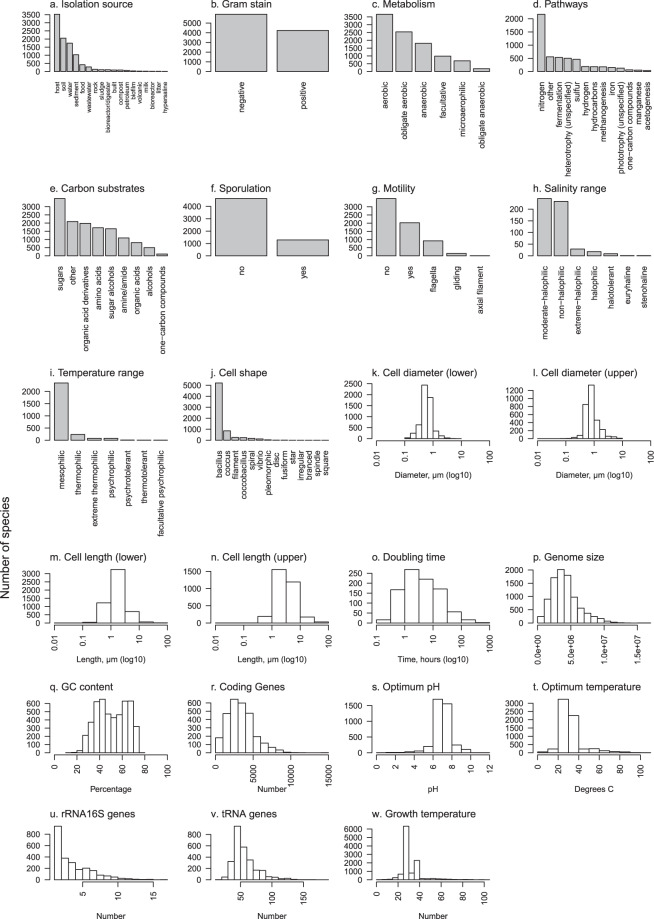
Graphical summaries of each of 23 traits in Online-only Table 2. Barplots are used for categorical traits and frequency histograms for continuous traits. Due to the high number of distinct metabolic pathways (>80) (**d**) and carbon substrates (>100) (**e**) included in this data, to simplify presentation each of these were grouped into major categories; pathways were grouped by the primary compound involved or distinct processes where no primary compound exists, and carbon substrates were grouped by chemical classification.

The scripted workflow was developed to reproducibly (a) prepare datasets to be merged; (b) combine datasets; (c) condense similar or the same traits into columns; and (d) condense rows into species based on either the NCBI taxonomy^17^ or the Genomic Taxonomy Database (GTDB) taxonomy^18^ (Fig. 1, Online-only Table 1). This workflow generated five data products^17^ for the 23 phenotypic, genomic and environmental traits shown in Online-only Table 2. The first two products are record level, which includes taxonomic levels below species (e.g., strain) and based on the NCBI taxonomy and GTDB taxonomy, respectively. A reference table was generated to track provenance of raw data through the workflow. The last two products are aggregated at species-level for the NCBI taxonomy and GTDB taxonomy, respectively. Trait coverage across the phylogenetic tree is shown in Fig. 2 and the trait distributions are shown in Fig. 3. Table 1 shows species-level trait data derived from original datasets.

**Table 1:** Summary of raw trait data points per source.

	amend-shock	bacdive-microa	campeelli	corkrey	edwards	engqvist	faprotax	fierer	genbank	gold	jemma-refseq	kegg	kremer	masonmm	mediadb	methanogen	microbe-directory	nielsensl	pasteur	patric	prochlorococcus	protraits	roden-jin	rrndb	silva
gram_stain	0	0	0	0	0	0	0	0	0	25,084	0	0	0	0	0	114	2,335	0	0	13,979	0	2,266	0	0	0
metabolism	0	1336	182	661	0	0	0	4,423	0	10,311	0	0	0	0	0	153	0	0	5,477	10,534	0	579	0	0	0
pathways	610	0	0	0	0	0	9,515	1,427	0	0	0	0	0	0	0	153	0	0	0	0	0	272	99	0	0
carbon_substrates	0	0	0	0	0	0	0	4,534	0	0	0	0	0	0	0	150	0	0	0	0	0	0	0	0	0
sporulation	0	0	0	0	0	0	0	3,322	0	7,258	0	0	0	0	0	0	1,564	0	0	4,174	0	2,738	0	0	0
motility	0	0	0	0	0	0	0	4,356	0	8,724	0	0	0	0	0	126	0	0	0	8,657	0	552	0	0	0
range_tmp	0	0	0	0	0	0	0	0	0	0	0	0	0	0	0	0	0	0	0	7,833	0	0	0	0	0
range_salinity	0	0	0	0	0	0	0	0	0	0	0	0	0	0	0	0	0	0	0	922	0	0	0	0	0
cell_shape	0	0	0	0	0	0	0	4,478	0	9,602	0	0	0	0	0	153	0	0	0	13,088	0	632	0	0	0
isolation_source	0	0	191	0	9	0	0	4,672	0	45,146	488	278	31	0	0	0	0	0	1,104	0	22	0	0	0	0
d1_lo	0	0	0	0	0	0	0	3,774	0	1,014	0	0	0	0	0	147	0	6	0	0	12	0	0	0	0
d1_up	0	0	0	0	0	0	0	926	0	708	0	0	0	0	0	147	0	0	0	0	7	0	0	0	0
d2_lo	0	0	0	0	0	0	0	3,794	0	1,028	0	0	0	0	0	148	0	0	0	0	3	0	0	0	0
d2_up	0	0	0	0	0	0	0	1,043	0	859	0	0	0	0	0	148	0	0	0	0	0	0	0	0	0
doubling_h	0	0	0	661	9	0	0	0	0	0	0	0	31	42	37	119	0	6	0	0	22	0	0	0	207
genome_size	0	0	0	0	0	0	0	0	11,344	77,307	1,727	4,664	0	0	0	0	0	0	0	12,311	0	0	0	0	0
gc_content	0	0	0	0	0	0	0	0	11,351	0	0	0	0	0	0	0	0	0	0	16,781	0	0	0	0	0
coding_genes	0	0	0	0	0	0	0	0	11,251	0	1,610	4,670	0	0	0	0	0	0	0	0	0	0	0	0	0
optimum_tmp	0	0	0	0	0	0	0	4,251	0	4,539	0	0	0	0	0	152	1,559	0	0	3,963	0	0	0	0	0
optimum_ph	0	0	0	0	0	0	0	3,429	0	0	0	0	0	0	0	148	994	0	0	0	0	0	0	0	0
growth_tmp	0	0	195	661	9	12,530	0	0	0	0	0	0	31	6	31	0	0	0	0	0	0	0	0	0	202
rRNA16S_genes	0	0	0	0	0	0	0	0	0	0	1,609	0	0	0	0	0	0	0	0	0	0	0	0	5,637	0
tRNA_genes	0	0	0	0	0	0	0	0	11,237	0	1,610	0	0	0	0	0	0	0	0	0	0	0	0	18	0
**Total data points:**	**610**	**1336**	**568**	**1,983**	**27**	**12,530**	**9,515**	**44,429**	**45,183**	**191,580**	**7,044**	**9,612**	**93**	**48**	**68**	**1,858**	**6,452**	**12**	**6,581**	**93,489**	**66**	**7,039**	**99**	**5,655**	**409**

### Prepare

The preparation steps removed unwanted columns from raw datasets, ensured standard trait (column) naming, and established that each record (row) had an NCBI taxon ID and reference. In cases where NCBI taxon IDs were not provided in the raw dataset, taxon mapping tables were created using the NCBI taxonomy API, which could retrieve IDs by fuzzy searches of name or accession number, depending on what was available^10,17^. In cases where the API did not resolve to a single taxon, the NCBI taxonomy browser was used to manually look-up parts of names in case of misspellings or name fragments (e.g., strain names that were truncated to species level). DOIs or full text citations were used for referencing where possible, but in some cases only NCBI BioProject or accession numbers were available and were used to track provenance instead. All changes in the preparation stage were scripted and commented in dataset-specific preparation scripts. Other dataset-specific steps included splitting number ranges into different components (e.g., 10-20 µm to 10 [min], 20 [max] and µm [unit]), and any general data translation issues (e.g., spreadsheet software issues that manipulated characters, dates, and other inconsistencies). Only the traits summarised in Online-only Table 2 were retained for the steps where data are combined (next).

### Combine

All the raw datasets were placed into a single sparse matrix with zero overlap (Fig. 1b). A column was added with the name of the dataset (Online-only Table 1) to keep track of dataset provenance. All columns containing referencing information (reference and reference type) and NCBI taxon IDs were moved into dedicated columns. The basic taxonomic hierarchy was mapped onto each row using either of the NCBI or GTDB taxonomies, which added columns for species, genus, family, order, class, phylum and superkingdom.

### Condense traits

Condensing trait data involved moving values for the same trait from different datasets into one column (Fig. 1c). The inherent assumption is that data for the same taxon from different datasets were observed independently (e.g., cell sizes for a given strain or species that occurred in multiple datasets were considered different observations, and so are included as multiple rows). This assumption had little influence on the data following the condense species step (next). During the condense traits step, columns with categorical values were mapped into a predefined nomenclature using manually defined lookup tables (e.g., sporulation values were mapped to either “yes” or “no”; Online-only Table 2).

Isolation source or habitat information for prokaryotes follows different schemes in different data sources, and often is unstructured, consisting of a string of words or sentences. With a view to making possible investigation of species and trait distributions across environments, we have developed for this data synthesis a scheme consisting of approximately 100 environment labels. The scheme is hierarchical using up to four levels of specificity, for example a one-term label is “host”, a two-term is “host_animal”, a three-term is “host_animal_endotherm”, and a four-term is “host_animal_endotherm_intestinal”). This allowed us to be relatively specific or relatively vague depending on the information available. To translate environment information into this new scheme, all columns in each data-source that contained environment information were concatenated into one comma-separated string, thus capturing as much information as was available in the data source. These concatenated strings were then manually translated into their most appropriate label in terms of our scheme and saved in a translation table. Given the large number of unique strings created in this way, only the most prevalent strings have at this stage been translated (>3,000), covering approximately 65% of the species in the species condensed dataset. These environmental labels were annotated with terms from the Environmental Ontology (ENVO) and stored in the “environments.csv” table in the GitHub project; however, ENVO annotations do not currently appear in the data products^19^ because most environmental terms required the union of multiple ENVO terms.

A step was also included to correct datum-specific errors. Some of these likely occurred during original data entry, such as wrong units or misspellings. Others were values that seemed surprising, and also stronger or newer evidence was available from other sources. These corrections were scripted as a translation table that contained the original dataset, taxon, trait and value where the error occurred, and then the new, corrected value as well as a comment and reference as to why the change was made (see Technical Validation). The condense trait step generated three files^19^: “condensed_traits_NCBI.csv”, “condensed_traits_GTDB.csv” and “references.csv”.

### Condense species

At this stage, rows in the dataset represented both strains and species, and each strain and species could have multiple replicate rows for a given trait. Because every row could be mapped to species (but not vice versa), data were aggregated at either the NCBI^10,17^ or GTDB^18^ species level. That is, all records for a given species, and strains of that species, were condensed into one record. All rows not resolved to species using these taxonomies were excluded (e.g., those with “sp.” instead of a recognised species name).

For numerical traits, aggregation consisted of calculating the average, standard deviation and number of records for a given species/trait combination. These derived values were saved as columns labelled by the trait name and then the trait name with “.stdev” and “.count” appended, respectively. The script for species condensation can be altered to calculate other derived values, like median, minimum, maximum, and so on.

For categorical traits, the majority rule was used, where terms for a given trait were tallied and the term with greater than 50% of the tally was assigned as the species aggregate. For binary categorical variables (e.g., gram stain, sporulation), and also cell shape, only the dominant term (>50% of total) was assigned and, in the case of ties, no term was assigned (i.e., the value was left blank). For categorical variables with multiple terms and levels of specificity (e.g., metabolism and motility), the following logic was employed:

- If no single term dominated, a simple logic was used to select the most appropriate term based on grouping of terms into main categories of resemblance (e.g., aerobic vs. anaerobic, motile vs. non-motile) and specificity level (e.g., “aerobic” was considered less specific than “obligate aerobic”; for motility, “yes” was considered less specific than “flagella”).
- If all terms belong to the same category, the most specific term was selected (e.g., “obligate aerobic” selected instead of “aerobic”).
- If all terms belong to the same category and all have the same level of specificity (e.g., “facultative aerobic” and “obligate aerobic”), the term is converted to its least specific form (i.e., “aerobic”).
- If terms belong to different categories (e.g., “aerobic” vs. “anaerobic”), then no term was assigned (i.e., the value was left blank).

Due to the hierarchical nature of the naming schemes for isolation sources, selecting the most representative term was done on a per-level basis. Each isolation source term potentially contained up to 4 levels of detail (e.g., level 1: host, level 2: animal, level 3: endotherm and level 4: blood). For each level (starting at level 1 and proceeding through levels 1 to 4), the occurrence of each term amongst all observations for a given species was counted, and the dominant term chosen and combined with the dominant term in the next level. If no dominant term could be found at a given level (not resolved), the process was stopped at that level. As such, an isolation source may contain 1 to 4 levels of information with increasing specificity.

Bergey’s Manual of Systematics of Archaea and Bacteria^11^ contains a large amount of useful phenotypic trait detail, such cell size, sporulation, gram, metabolism and more, across the whole of Archaea and Bacteria, but is not stored as a dataset. Therefore, this data source was used at the final stage of the species condense step to fill in data gaps, especially for traits that were easily extractable using text matching (e.g., cell size and metabolism; see scripted workflow for details). The condense species step generated two files^19^: “condensed_species_NCBI.csv” and “condensed_species_GTDB.csv”.

## Data Records

1. “condensed_traits_NCBI.csv”: A trait condensed data record containing all focal trait data (Online-only Table 2) from original datasets using the NCBI taxonomy^19^. Rows represent strain- or species-level measurements, and there can be more than one row per taxon. On the whole, this is a strain-level, non-aggregated data record.
2. “condensed_traits_GTDB.csv”: Same as “condensed_traits_NCBI.csv” but using the GTDB taxonomy^19^. This trait condensed data record is smaller, because the GTDB protocol does not accept all NCBI taxa.
3. “references.csv”: A table containing reference information for the data^19^. Each row in the trait condensed data (“condensed_traits_NCBI.csv” and “condensed_traits_GTDB.csv”) has a unique ID that points to a reference in the reference table for that particular data record. Species condensed data (below) have multiple reference IDs.
4. “condensed_species_NCBI.csv”: A species condensed data record contained all focal traits (Online-only Table 2) aggregated so that there is one row per NCBI-defined species^19^.
5. “condensed_species_GTDB.csv”: Same as “condensed_species_NCBI.csv” but using the GTDB taxonomy^19^. However, this species condensed data record is smaller, because the GTDB protocol does not accept all NCBI taxa.

## Technical validation

Approximately 80% of the time spent developing this bacteria and archaea trait data pipeline was consumed by searching for and fixing errors and inconsistencies in the raw datasets that were ultimately combined. When inconsistencies across datasets could not be resolved, the data were removed. These fixes necessarily involved human judgment, hence the large time expense. All fixes to datasets have been recorded into a data correction table (in “data/conversion_tables/data_corrections.csv”) that is implemented by the script so that the decision-making process is transparent. In addition to basic error checking (e.g., looking at unique lists of controlled terms, removing whitespace, etc.), we paid particular attention to outliers, which sometimes (though certainly not always) turned out to be problematic. We located outliers by inspecting distributions of the continuous traits, and also bivariate plots (e.g., by sorting residuals from model fits), or boxplots where one variable was categorical. Users who find and wish to correct further errors, or who wish to apply a different judgment about anomalous and outlier traits, can readily implement this through the same data correction and other data translation tables in the GitHub repository.

## Usage Notes

The data records are available at figshare^19^. The script that generated the data records is available at GitHub (https://github.com/bacteria-archaea-traits/bacteria-archaea-traits/releases/tag/v1.0.0). Two large files were not included with the GitHub project: the NCBI taxonomy translation table and PATRIC dataset. These files are automatically downloaded to their correct directories the first time the workflow script is run. If download problems occur, instructions for where to place these large files manually can be found in the project readme file.

Please note that several of the raw datasets entering into the workflow were sourced from dynamic, growing databases (see Online-only Table 1). Therefore, users of the Data Records may consider obtaining fresh versions of the different sources from the links or data providers in Online-only Table 1, and then re-applying the scripted workflow to build an updated data synthesis. Additionally, the datasets we merge contain additional traits that we do not collect in our workflow, given our broader research goals. Adding these traits requires adjusting the project settings and editing dataset specific preparation files. Instructions for doing so are in the project readme file and dataset specific readme files (“data/raw”). Translation tables created to map trait variables, including isolation source, are in the “data/conversion_tables” directory. Additional quality control will be necessary following the addition of new or updated datasets and traits to the workflow.

We encourage other groups who update or add new data sources to this data product to do so using our procedure outlined in the Methods (above) and in more detail at the GitHub project readme. This project uses GitHub’s standard fork and pull request workflow, which is well documented at GitHub. Such changes would follow this general pattern:

- Forking the GitHub project.
- Updating the existing or adding the new dataset in its raw form to the “data” repository.
- Writing a data preparation script (“R/preparation”), which includes appending NCBI taxon IDs if not already in the dataset.
- Identifying the traits to be merged (“R/settings.R”), and writing a conversion table if the trait is not in the same units of categories as the present dataset version (“data/conversion_tables”).
- Looking for outliers and other errors, which can be removed or altered using the corrections table (“data/conversion_tables/data_corrections.csv”)
- Running and testing the merger (“workflow.R”).
- Submitting a pull request via GitHub, at which point we will review and test the changes.
- Once the pull request is accepted, the project version will be updated.

## Data Availability

The complete data workflow was scripted in the programming language R (https://www.R-project.org) and instructions for generating the merged data sets accompanying this data descriptor can be found at GitHub (https://github.com/bacteria-archaea-traits/bacteria-archaea-traits/releases/tag/v1.0.0).

## Online-only Tables

**Online-only Table 1:** Summary of original datasets.

	Short name	Name	Source	Direct access	Format
1	Amend-shock	Energetics of overall metabolic reactions of thermophilic and hyperthermophilic Archaea and Bacteria^21^	doi.org/10.1111/j.1574-6976.2001.tb00576.x	Yes	pdf
2	Bacdive-microa	The Bacterial Diversity Metadatabase^22^	bacdive.dsmz.de	Yes	txt
3	Bergeys	Bergey’s Manual of Systematic Bacteriology^11^	doi.org/10.1002/97811189 60608	No	pdf
4	Campedelli	Genus-wide assessment of antibiotic resistance in Lactobacillus spp.^23^	doi.org/10.1128/AEM.01738-18	Yes	pdf
5	Corkrey	The Biokinetic Spectrum for Temperature^24^	doi.org/10.1371/journal.po ne.0153343.s004	Yes	txt
6	Edwards	Nutrient utilization traits of phytoplankton^25^	doi.org/10.6084/m9.figshar e.c.3307917	Yes	txt
7	Engqvist	Correlating enzyme annotations with a large set of microbial growth temperatures reveals metabolic adaptations to growth at diverse temperatures^26^	doi.org/10.5281/zenodo.11 75609	Yes	tsv
8	Faprotax	Functional Annotation of Prokaryotic Taxa (FAPROTAX)^27^	pages.uoregon.edu/slouca/LoucaLab/archive/FAPROTAX/	Yes	txt
9	Fierer	International Journal of Systematic and Evolutionary Microbiology (IJSEM) phenotypic database^28^	doi.org/10.6084/m9.figshar e.4272392.v3	Yes	txt
10	Genbank	Annotated DNA sequences^10^	www.ncbi.nlm.nih.gov/gen bank	Yes	txt
11	GOLD	Genomes OnLine Database^29^	gold.jgi.doe.gov/index	No	txt
12	Jemma-refseq	Refseq data extraction based on custom text-extraction code^10^	www.ncbi.nlm.nih.gov/refs eq	No	txt
13	KEGG	Kyoto Encyclopedia of Genes and Genomes^30^	www.genome.jp/kegg	No	html
14	Kremer	Temperature- and size-scaling of phytoplankton population growth rates: Reconciling the Eppley curve and the metabolic theory of ecology^31^	doi.org/10.1002/lno.10523	Yes	txt
15	Masonmm	Maximal growth rates of various bacteria under optimal conditions^32^	www.ncbi.nlm.nih.gov/pmc/articles/PMC545149	No	pdf
16	MediaDB	Chemically-defined growth conditions^33^	mediadb.systemsbiology.ne t	Yes	sql
17	Metanogen	PhyMet2^34,35^	metanogen.biotech.uni.wro c.pl/	Yes	txt
18	Microbe- Directory	Annotation for metagenomic taxonomic analyses^36^	microbe.directory/ (github.com/microbe- directory/microbe- directory)	Yes	txt/sql
19	Nielsensl	Size-dependent growth rates in eukaryotic and prokaryotic algae exemplified by green algae and cyanobacteria: comparisons between unicells and colonial growth forms^37^	doi.org/10.1093/plankt/fbi134	No	txt
20	Pasteur	Centre de Ressources Biologiques de l’Institut Pasteur - Microorganism biobank catalogue	catalogue- crbip.pasteur.fr/recherche_ catalogue.xhtml	Yes	txt
21	PATRIC	Pathosystems resource integration center^38^	www.patricbrc.org	Yes	txt
22	Prochlorococcus	Various marine cyanobacteria doubling times	Lisa Moore (co-author)	No	txt
23	ProTraits	Phenotypes assigned to microbes using machine learning and text mining^39^	protraits.irb.hr (95% precision dataset used)	Yes	txt
24	Roden-jin	Thermodynamics of Microbial Growth Coupled to Metabolism of Glucose, Ethanol, Short-Chain Organic Acids, and Hydrogen^40^	doi.org/10.1128/AEM.02425-10	Yes	pdf
25	RRDN	Ribosomal RNA operons^41^	rrndb.umms.med.umich.ed u/static/download/	Yes	tsv
26	Silva	Growth data for ecological metagenomics^42^	doi.org/10.1371/journal.pg en.1000808.s005	Yes	doc

**Online-only Table 2:** Summary of microbe traits including information about measurements and statistics about taxon coverage in the accompanying data records.

	Trait name	Description	Measurement type	Units or categoric al terms	Trait category	Number of observations (of possible 169743)	Number of NCBI species (of possible 14884)	Percent of NCBI species (%)
1	Isolation source	Where the microbe was sourced from	Textual description	(Multiple, hierarchical)	Habitat	51977	9776	65.7
2	Gram stain	Gram positive or negative	Binary	+, −	Physiological	44196	10141	68.1
3	Metabolism	Oxygen usage	Categorical	Obligate aerobic, Aerobic, Facultative, Microaerophilic, Anaerobic, Obligate anaerobic	Physiological	34585	9869	66.3
4	Pathways	List of metabolic pathways undertaken	Categorical	(Multiple, hierarchical)	Physiological	12076	3822	25.7
5	Carbon substrate	List of carbon substrates that can be utilised	Categorical	(Multiple, hierarchical)	Physiological	4684	4151	27.9
6	Sporulation	Can produce spores	Binary	Yes, No	Physiological	19080	5916	39.7
7	Motility	Capacity to move	Categorical	Yes, No, Flagella, Gliding, Axial filament	Physiological	22763	6596	44.3
8	Salinity range	Coarse environmental preference	Categorical	Low, Moderate, High, Extreme	Environmental	922	536	3.6
9	Temperature range	Coarse environmental preference	Categorical	Low, Medium, High, Extreme	Environmental	8799	2753	18.5
10	Cell shape	The typical shape of cells	Categorical	(Multiple)	Morphological	28326	6891	46.3
11	Cell diameter (lower)	The lower range of cell diameters	Length	µm	Morphological	4980	5726	38.5
12	Cell diameter (upper)	The upper range of cell diameters	Length	µm	Morphological	1799	3102	20.8
13	Cell length (lower)	The lower range of cell length, where applicable based on shape	Length	µm	Morphological	5000	5294	35.6
14	Cell length (upper)	The upper range of cell length, where applicable based on shape	Length	µm	Morphological	2062	3146	21.1
15	Doubling time	Growth rate estimate based on doubling number of cells	Time	Hours	Physiological	1134	917	6.2
16	Genome size	Number of base pairs making up the genome	Count	Base pairs	Genomic	108558	9115	61.2
17	GC content	Percentage of base pairs that are guanine or cytosine	Ratio	Percentage	Genomic	29382	4832	32.5
18	Coding genes	The number of coding genes	Count	Base pairs	Genomic	17531	2791	18.8
19	Optimum pH	The preferred pH in which to live	pH	pH	Physiological	4604	3927	26.4
20	Optimum temperature	The preferred temperature in which to live	Temperature	Degrees C	Physiological	15193	6517	43.8
21	16S rRNA genes	The number of 16s rRNAgenes	Count	Base pairs	Genomic	7246	2430	16.3
22	tRNA genes	The number of tRNA genes	Count	Base pairs	Genomic	12865	2742	18.4
23	Growth temperature	Temperature for specific growth measurements	Temperature	Degrees C	Contextual (for doubling time)	13665	11265	75.7
